# Supplementary material for: Diversity-Oriented Synthesis (DOS) Towards Calcitriol Analogs with Sulfur-Containing Side Chains
Source: Int J Mol Sci. 2025 Jun 28;26(13):6266. doi: 10.3390/ijms26136266 (PMC12250139; doi:10.3390/ijms26136266)
Supplement: Supplementary file 1 [file ijms-26-06266-s001.zip › ijms-3686783-supplementary.pdf]

Supporting information

Diversity Oriented Synthesis (DOS) towards Calcitriol analogs with sulfur–containing side chains

Uxía Gómez-Bouzó, Generosa Gómez * and Yagamare Fall *

Departamento de Química Orgánica and Instituto de Investigación Sanitaria Galicia Sur (IISGS), Campus Lagoas Marcosende, Universidad de Vigo, 36310 Vigo, Spain.

Table S1. Crystal data and structure refinement for 32093801_0m_a.

Identification code	32093801_0m_a	
Empirical formula	C28 H54 O4 S Si2	
Formula weight	542.95	
Temperature	100.00 K	
Wavelength	0.71073 Å	
Crystal system	Orthorhombic	
Space group	P212121	
Unit cell dimensions	a = 6.9376(12) Å	α= 90°
	b = 13.3487(16) Å	β= 90°
	c = 34.618(4) Å	γ = 90°
Volume	3205.9(8) Å3	
Z	4	
Density (calculated)	1.125 Mg/m3	
Absorption coefficient	0.204 mm-1	
F(000)	1192	
Crystal size	0.254 x 0.102 x 0.053 mm3	
Theta range for data collection	1.927 to 28.354°.	
Index ranges	-9<=h<=9, -16<=k<=17, -46<=l<=46	
Reflections collected	54354	
Independent reflections	7981 [R(int) = 0.0564]	
Completeness to theta = 25.242°	99.8 %	
Absorption correction	Semi-empirical from equivalents	
Max. and min. transmission	0.7457 and 0.5851	
Refinement method	Full-matrix least-squares on F2	
Data / restraints / parameters	7981 / 0 / 328	
Goodness-of-fit on F2	1.077	
Final R indices [I>2sigma(I)]	R1 = 0.0479, wR2 = 0.1299	
R indices (all data)	R1 = 0.0498, wR2 = 0.1310	
Absolute structure parameter	0.04(3)	
Extinction coefficient	n/a	
Largest diff. peak and hole	0.827 and -0.341 e.Å-3	
